# Association of dietary inflammatory index and dietary oxidative balance score with gastrointestinal cancers in NHANES 2005–2018

**DOI:** 10.1186/s12889-024-20268-4

**Published:** 2024-10-09

**Authors:** Yu Chang, Chanjiao Yu, Xianyu Dai, Haibo Sun, Tongyu Tang

**Affiliations:** The First Hospital of Jilin University, No.1 Xinmin Street, Changchun, 130012 China

**Keywords:** Gastrointestinal cancers, Dietary inflammatory index (DII), Dietary oxidative balance score (DOBS), NHANES

## Abstract

**Background&Aims:**

Gastrointestinal (GI) cancers, including gastric, liver, esophageal, pancreatic, and colorectal cancers, represent significant global health burdens. Emerging evidence suggests that dietary patterns, particularly their inflammatory and oxidative properties, may influence cancer risk. The Dietary Inflammatory Index (DII) and Dietary Oxidative Balance Score (DOBS) assess the inflammatory and oxidative effects of diets, respectively. This study aims to explore the association between DII, DOBS, and the combined risk of GI cancers, and investigates the potential mediating roles of serum albumin and red cell distribution width (RDW).

**Methods:**

Data from 26,320 participants in the NHANES 2005–2018 cycles were analyzed. DII was calculated based on 28 dietary components, and DOBS included 17 nutrients (3 pro-oxidants and 14 antioxidants). Logistic regression models assessed the associations between DII, DOBS, and GI cancers. Restricted cubic spline (RCS) models examined dose-response relationships. Mediation analysis evaluated the roles of serum albumin and RDW. Subgroup analyses explored interactions with demographic and health-related factors.

**Results:**

Higher DII was associated with increased GI cancer risk (OR: 1.26, 95% CI: 1.07–1.49 per unit increase), while higher DOBS was associated with reduced risk (OR: 0.90, 95% CI: 0.76–0.99 per unit increase). RCS analysis indicated a significant nonlinear relationship between DII and GI cancer risk. Serum albumin and RDW partially mediated the associations between DII, DOBS, and GI cancers. Subgroup analyses showed stronger associations for DII among certain demographics, and significant interactions were found between DII and BMI. For DOBS, significant interactions were observed with age and BMI.

**Conclusion:**

This study reveals significant associations between dietary inflammatory and oxidative balance scores and GI cancer risk. Higher DII is linked to increased risk, while higher DOBS is protective. The mediating roles of serum albumin and RDW provide insights into underlying mechanisms. These findings underscore the potential of dietary modifications in GI cancer prevention and management, emphasizing the importance of anti-inflammatory and antioxidant-rich diets.

**Supplementary Information:**

The online version contains supplementary material available at 10.1186/s12889-024-20268-4.

## Introduction

Gastrointestinal (GI) cancers include gastric cancer, liver cancer, esophageal cancer, pancreatic cancer, and colorectal cancer [1].According to the World Health Organization (WHO), colorectal cancer accounts for over a quarter of global cancer incidence and one-third of cancer-related deaths [2]. Globally, gastric cancer is considered the second most common cause of cancer-related deaths [3]. While smoking and alcohol consumption are the most common risk factors associated with cancer development, there is increasing consensus on dietary habits as a risk factor for GI cancers [1]. Considering that dietary factors are still the main drivers of the global burden of chronic diseases such as cancer, diet is a key modifiable target for reducing the risk of chronic diseases [4]. Therefore, dietary modifications for the prevention of GI cancers play a crucial role in reducing cancer risk.

Diet can influence cancer risk through various mechanisms, including modulation of the gut microbiome, oxidative stress, and energy balance [5]. The basis of these mechanisms lies in dietary patterns and the potential pro-inflammatory or anti-inflammatory properties of individual dietary components. The Dietary Inflammatory Index (DII) and Dietary Oxidative Balance Score (DOBS) are indicators based on the inflammatory and oxidative effects of nutrients in the diet, used to assess the impact of dietary components on bodily inflammation and oxidative stress. DII is based on 45 food parameters, including individual nutrients (such as omega-3 fatty acids), compounds (such as flavonoids), and foods (such as garlic and ginger), identified for their anti-inflammatory or pro-inflammatory properties [6]. DOBS comprises a composite score of 14 antioxidants and 3 pro-oxidants in dietary components. Generally, a higher DOBS score indicates a stronger antioxidant capacity of the diet. Diets with high DII and low DOBS typically involve high sugar, fat, salt, and cholesterol, promoting inflammation and oxidative stress. Conversely, diets with low DII and high DOBS often imply high intakes of vegetables, fruits, protein, and dietary fiber, which can reduce levels of inflammation and oxidative stress. Increasingly, studies have reported associations between high DII or low DOBS and the risk of various diseases, including diabetes, cardiovascular diseases, and cancer [7, 8].

Serum albumin is an important biomarker involved in the pathogenesis of GI cancers through its roles in inflammation and antioxidation. As the body’s principal plasma protein, it not only reflects the nutritional status but also indicates systemic inflammatory states [9]. Low protein levels are often closely associated with malnutrition and chronic inflammatory conditions, which are established risk factors for GI cancers [10]. Red cell distribution width (RDW) reflects the heterogeneity of red blood cell volumes and serves as an important marker of chronic inflammation. Elevated RDW is closely associated with oxidative stress and has been linked to inflammatory markers in conditions such as Crohn’s disease, rheumatoid arthritis, and cardiovascular diseases [11–13]. However, whether serum albumin and RDW mediate the relationship between DII, DOBS, and GI cancers has not been sufficiently explored.

The aim of this study was to further investigate the correlation between DII, DOBS, and GI cancers in a nationally representative sample of American adults. We constructed logistic regression models and plotted restricted cubic splines (RCS) to assess the relationship between them. Additionally, we explored the potential mediating roles of serum albumin and RDW in the relationship between DII, DOBS, and GI cancers. Understanding the roles of DII and DOBS along with these biomarkers in the development of GI cancers may provide valuable insights for the prevention and management of GI cancers.

## Methods

### Study population

In this study, we utilized data from the NHANES database spanning from 2005 to 2018. The NHANES database is a stratified, national, large-scale cross-sectional survey conducted in two-year cycles, aimed at assessing the nutritional and health status of adults and children in the United States. The NHANES database includes modules on demographics, diet, physical examinations, laboratory tests, and questionnaires. More detailed information about NHANES can be obtained from its official website at https://www.cdc.gov/nchs/nhanes/index.htm.

**Fig. 1:**
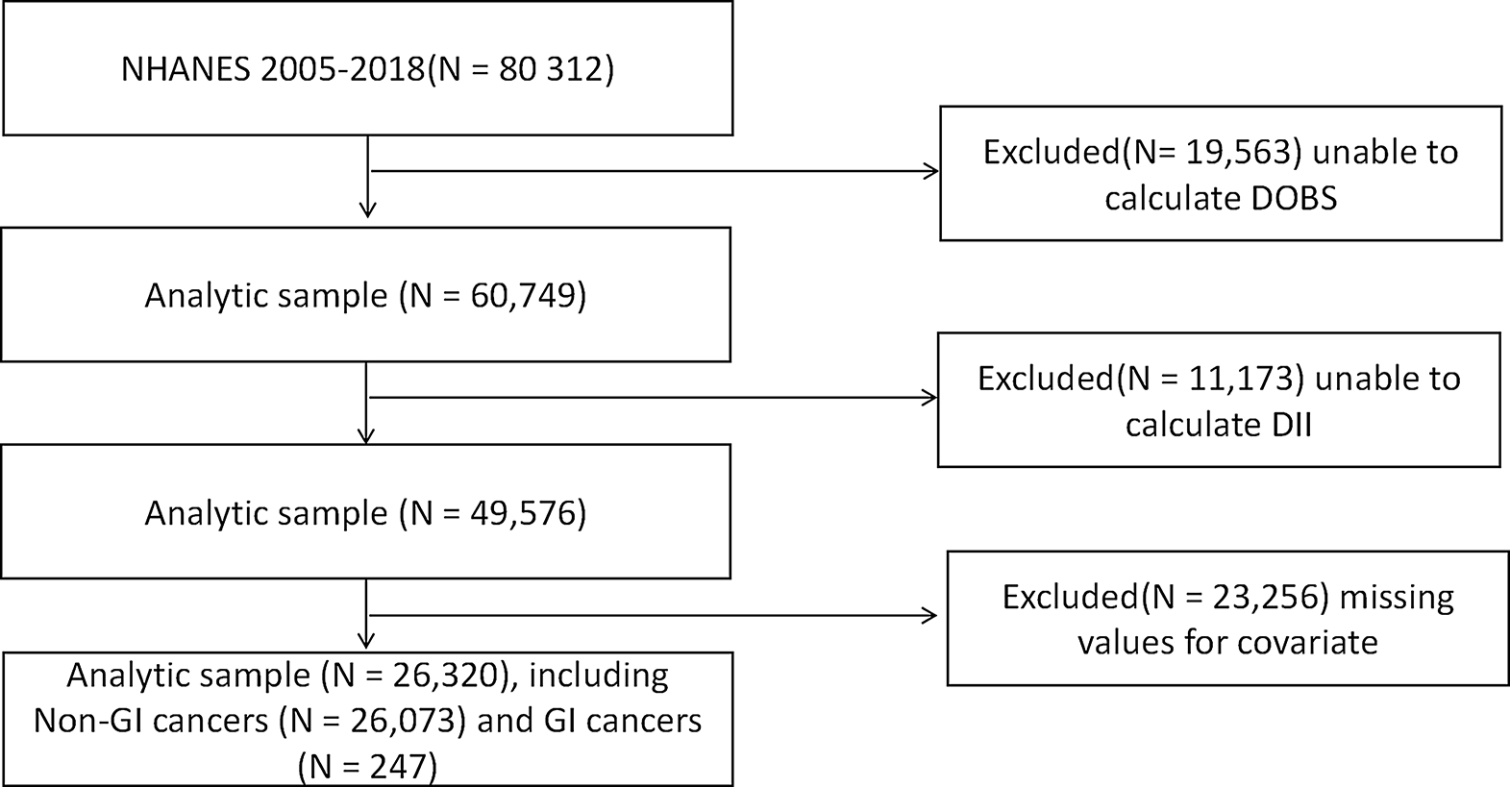
Flowchart for the Study Population: From NHANES 2005–2018

This study collected data from a total of 80,312 participants. Of these, 19,563 participants were excluded due to an inability to calculate DOBS. Additionally, 11,173 participants were excluded because DII could not be calculated. A further 23,256 participants were excluded due to missing other covariates. Ultimately, 26,320 participants were included in this study. Of these, 13,598 were female participants and 12,722 were male. For more specific details, refer to Fig. 1.

### Accessment of DII and DOBS

Dietary data for this study were primarily collected by the Nutrition Methods Working Group through face-to-face and telephone interviews at Mobile Examination Centers (MEC) using two 24-hour dietary recall questionnaires. DII was calculated based on 28 dietary components, following previously published computation protocols. Six different inflammatory markers were used to assess various levels of inflammation. Components that significantly increased the levels of interleukins (IL)-1β, IL-6, C-reactive protein (CRP), tumor necrosis factor (TNF)-α, or significantly decreased the levels of IL-4 and IL-10 were scored as “+1”. Conversely, components that decreased the levels of IL-1β, IL-6, CRP, and TNF-α, or increased the levels of IL-4 and IL-10 were scored as “-1”. If a dietary component did not alter the levels of inflammatory markers, it was deemed to have no inflammatory properties and was scored as “0”. In the overall inflammatory index, positive scores indicate pro-inflammatory potential, whereas negative scores indicate anti-inflammatory potential. The higher the presence of pro-inflammatory potential foods in the diet, such as carbohydrates and saturated fats, the higher the DII value will be. Conversely, the higher the content of anti-inflammatory foods in the diet, such as fruits, green vegetables, and whole grains, the lower the DII value [6]. Details on the dietary components related to DII are provided in Supplementary Table 1. In this study, we selected 28 out of 45 dietary components to calculate the DII. The formula for calculating DII is: (Daily intake of a dietary component - Global daily average intake of the component) / Standard deviation of the global daily intake of the component * Overall inflammatory effect score of the dietary component. Therefore, the sum of the DIIs of the 28 dietary components represents the participant’s overall DII.

In this study, we identified pro-oxidant and antioxidant factors in the diet based on previously published research. The calculation of DOBS includes 17 nutrients, comprising 3 pro-oxidants (total fats, alcohol, iron) and 14 antioxidants (dietary fiber, carotenoids, vitamin B2, niacin, vitamin B6, folate, vitamin B12, vitamin C, vitamin E, calcium, magnesium, zinc, copper, selenium). After categorizing continuous dietary variables into tertiles, antioxidant scores are assigned from 1 to 3, and pro-oxidant scores are inversely assigned from 3 to 1. For alcohol intake, heavy drinkers are defined as > 30 g/day for men and > 15 g/day for women, moderate drinkers as 0–30 g/day for men and 0–15 g/day for women, and non-drinkers as 0 g/day [14]. Detailed information about DOBS components can be found in Supplementary Table 2. The sum of the scores for the 17 dietary components constitutes a participant’s DOBS.

### Assessment of gastrointestinal cancers

Identification of gastrointestinal cancers was determined by accurate responses to the survey question “What kind of cancer” to ascertain if participants had GI cancers. Responses outside this question were used to determine that participants did not have GI cancers. In this study, we selected six types of GI cancers from the NHANES database: esophageal cancer, gastric cancer, liver cancer, pancreatic cancer, colon cancer, and rectal cancer as outcome variables. For more specific details, refer to the official website: https://wwwn.cdc.gov/Nchs/Nhanes/2005-2006/MCQ_H.htm#MCQ230a.

### Accessment of serum albumin and RDW

In the NHANES database, participants’ fasting serum samples were stored under appropriate frozen conditions (–30 °C) until they were sent to the National Center for Environmental Health for analysis. Serum albumin concentrations were measured by trained researchers using the bromocresol purple dye-binding method. Additionally, professional researchers performed complete blood counts on blood specimens using automated hematology analyzers based on the Beckman Coulter counting and quantification method. This provided the red blood cell distribution width (RDW) data for all participants. More details can be seen in https://wwwn.cdc.gov/Nchs/Nhanes/.

### Covariates

To more accurately assess the relationships between DII, DOBS, and GI cancers, we referred to previously published studies and constructed a Directed Acyclic Graph (DAG) based on the following variables, to visually summarize the relationships among these variables, DII, DOBS, and GI cancers [15–17]. Age, sex, educational attainment (less than high school, high school, college or above), ethnicity (Mexican American, Non-Hispanic Black, Non-Hispanic White, Other Race), alcohol consumption (yes/no), smoking status (yes/no), poverty-income ratio (PIR) (< 1, 1–3, ≥ 3), physical activity (moderate, vigorous, and other), hypertension (yes/no), diabetes (yes/no). Alcohol consumption was defined as drinking at the time of the survey or consuming more than 12 alcoholic drinks in a lifetime. Smoking was defined as smoking at the time of the survey or having smoked fewer than 100 cigarettes in a lifetime. poverty-income ratio (PIR) refers to the ratio of monthly household income to a poverty threshold specific to household size. Hypertension was defined as a self-reported diagnosis of hypertension, elevated average blood pressure (systolic ≥ 130 and/or diastolic ≥ 85 mm Hg), or current use of antihypertensive medication. Diabetes was defined as meeting any of the following conditions: (1) self-reported diagnosis of diabetes; (2) current use of insulin or hypoglycemic medication; (3) fasting blood glucose > 126 mg/dL, oral glucose tolerance test (OGTT) ≥ 200 mg/dL, glycated hemoglobin (HbA1c) ≥ 6.5%. More details can be seen in Supplementary Figs. 1–2.

### Statistical analysis

Considering the complex sampling design of NHANES, this study utilized weighted samples from NHANES and employed SAS survey procedures to calculate the total sample, ensuring nationally representative estimates. In this study, continuous variables were described using means (standard deviations [SD]) and categorical variables by counts (percentages). Differences in continuous variables between the GI cancers group and the non-GI cancers group were analyzed using t-tests and Wilcoxon rank-sum tests, depending on the data distribution. Categorical variables between the two groups were analyzed using Chi-square tests. Multiple logistic models were constructed to assess the association between DII, DOBS, and GI cancers. Model 1 was unadjusted for any variables. Model 2 was adjusted for sex, age, and ethnicity. Model 3 further adjusted for smoking status, alcohol consumption, educational attainment, PIR, physical activity, hypertension, and diabetes. A RCS model was used to evaluate the dose-response relationship between DII, DOBS, and GI cancers. The model included four knots at the 5th, 35th, 65th, and 95th percentiles of observed values (with the 5th percentile as the reference). Mediation analysis models were developed to determine whether serum albumin and RDW mediated the relationship between DII, DOBS, and GI cancers. Total effect (TE) represents the direct relationship between DII, DOBS, and GI cancers, uninfluenced by mediating factors. Indirect effect (IE) refers to the impact of DII, DOBS on GI cancers mediated through Serum Albumin and RDW. Direct effect (DE) represents the impact of DII, DOBS on GI cancers after controlling for Serum Albumin and RDW levels. A significant IE indicates a significant mediation effect, calculated as the proportion of mediation IE/TE*100%. Additionally, we conducted subgroup analyses across different ages, genders, ethnicities, educational levels, BMI, PIR, hypertension, and diabetes. The significance of interactions was assessed using the p-values of the product terms between DII, DOBS, and the aforementioned stratifying factors. We assessed the distribution of DII and DOBS using descriptive analyses, histograms, and the Shapiro-Wilk test for normality. Based on these assessments, non-parametric methods were applied in subsequent analyses to accommodate any deviations from normality. Besides, we conducted Spearman correlation analysis to assess the relationship between DII and DBOS, considering both as continuous variables. The relationship was further visualized using a scatter plot with a fitted regression line to illustrate the association between these dietary scores.

All statistical analyses and graphical representations were conducted using R software (version 4.3.3) and RStudio. Statistical tests were two-sided, and a p-value < 0.05 was considered statistically significant.

## Results

### Baseline characteristics

Baseline characteristics of participants are shown in Table 1. A total of 26,320 participants were included in this study, of which 26,073 did not have GI cancers, while 247 participants had GI cancers. This study included six types of gastrointestinal cancers, including colon cancer, rectal cancer, esophageal cancer, gastric cancer, liver cancer, and pancreatic cancer. Detailed information on cancer types and their distribution can be found in Supplementary Table 3. Compared to participants without GI cancers, those with GI cancers were more likely to be older, have higher DII levels, lower DOBS levels, be smokers, drink alcohol, have an education level below high school, belong to the Non-Hispanic White race, engage in moderate physical activity, and have a higher prevalence of hypertension and diabetes. Additionally, they exhibited lower albumin levels and higher RDW levels.

**Table 1:** Baseline characteristic of the study population

Variable	Total (*n* = 26320)	Non GI cancers (*n* = 26073)	GI cancers (*n* = 247)	*P*-value
Age (Mean ± SD)	49.76 ± 17.70	49.58 ± 17.65	68.45 ± 12.30	< 0.001
DII				< 0.010
Tertile 1	8773(33.33)	8710(33.41)	63(25.51)	
Tertile 2	8773(33.33)	8695(33.35)	78(31.58)	
Tertile 3	8774(33.34)	8668(33.25)	106(42.91)	
DOBS				< 0.010
Tertile 1	8543(32.46)	8438(32.36)	105(42.51)	
Tertile 2	8931(33.93)	8861(33.99)	70(28.34)	
Tertile 3	8846(33.61)	8774(33.65)	72(29.15)	
Smokers (%)				< 0.001
Yes	14,335(54.46)	14,237(54.60)	98(39.68)	
No	11,985(45.54)	11,836(45.40)	149(60.32)	
Drinking status (%)				0.050
Yes	7248(27.54)	7166(27.48)	82(33.20)	
No	19,072(72.46)	18,907(72.52)	165(66.80)	
Education Level (%)				0.040
Low high school	5807(22.06)	5736(22.00)	71(28.74)	
High school	6083(23.11)	6030(23.13)	53(21.46)	
College or above	14,430(54.83)	14,307(54.87)	123(49.80)	
Sex (%)				0.120
Female	13,598(51.66)	13,483(51.71)	115(46.56)	
Male	12,722(48.34)	12,590(48.29)	132(53.44)	
Race/ethnicity (%)				< 0.001
Mexican American	3847(14.62)	3835(14.71)	12( 4.86)	
Non-Hispanic Black	5566(21.15)	5514(21.15)	52(21.05)	
Non-Hispanic White	12,145(46.14)	11,988(45.98)	157(63.56)	
Other Race	4762(18.09)	4736(18.16)	26(10.53)	
Physical activity (%)				< 0.010
Moderate	14,460(54.94)	14,301(54.85)	159(64.37)	
Vigorous	6179(23.48)	6128(23.50)	51(20.65)	
Inactive	5681(21.58)	5644(21.65)	37(14.98)	
Hypertension (%)				< 0.001
No	16,700(63.45)	16,619(63.74)	81(32.79)	
Yes	9620(36.55)	9454(36.26)	166(67.21)	
Diabetes (%)				< 0.001
No	22,921(87.09)	22,740(87.22)	181(73.28)	
Yes	3399(12.91)	3333(12.78)	66(26.72)	
Poverty Income Ratio (%)				0.070
< 1	5245(19.93)	5201(19.95)	44(17.81)	
1–3	11,008(41.82)	10,887(41.76)	121(48.99)	
≥ 3	10,067(38.25)	9985(38.30)	82(33.20)	
BMI (kg/m^2^)				0.070
< 20	1142( 4.34)	1136( 4.36)	6( 2.43)	
20–25	6247(23.73)	6193(23.75)	54(21.86)	
25–30	8654(32.88)	8555(32.81)	99(40.08)	
≥ 30	10,277(39.05)	10,189(39.08)	88(35.63)	
Alb (Mean ± SD) (g/dl)	4.21 ± 0.36	4.21 ± 0.36	4.12 ± 0.37	< 0.001
RDW (Mean ± SD)	13.30 ± 1.36	13.29 ± 1.35	13.88 ± 1.99	< 0.001

*Notes* DII, Dietary Inflammatory Index; DOBS, Dietary Oxidative Balance Score; SD, Standard Deviation; BMI, Body Mass Index; Alb, Albumin; RDW, Red Cell Distribution Width

As shown in Supplementary Table 4, participants were grouped according to tertiles of the DII. Compared to the lowest tertile of DII, participants in the highest tertile were more likely to be female, have a BMI of 30 or above, have an educational level of high school or below, be non-Hispanic Black, drinkers, have a PIR < 3, engage in moderate physical activity, have hypertension, diabetes, lower plasma albumin levels, and higher RDW levels. As shown in Supplementary Table 5, compared to the lowest tertile of DOBS, participants in the highest tertile were more likely to be male, younger, have a BMI between 20 and 30, have an educational level of college or above, be non-Hispanic White, non-drinkers and non-smokers, have a PIR > 3, engage in inactive physical activities, not have hypertension or diabetes, have higher serum albumin levels, and lower RDW levels.

### Association between DII, DOBS and gastrointestinal cancers

As shown in Table 2, in Model 2, a continuous DII was significantly positively associated with the risk of gastrointestinal cancers (OR 1.30, 95% CI 1.11 to 1.53, *P* = 0.001). Compared to the lowest tertile of DII, the third tertile showed a 68% increased risk of gastrointestinal cancers (OR 1.68, 95% CI 1.22 to 2.33, *P* = 0.001). In Model 3, a continuous DII was also significantly positively associated with the risk of gastrointestinal cancers (OR 1.26, 95% CI 1.07 to 1.49, *P* = 0.007). Compared to the lowest tertile of DII, the third tertile showed a 58% increased risk of gastrointestinal cancers (OR 1.58, 95% CI 1.13 to 2.21, *P* = 0.007).

**Table 2:** The relationship between DII, DOBS and gastrointestinal cancers

	No. cases/participants	Model 1 OR (95% CI)	*P*-value	Model 2 OR (95% CI)	*P*-value	Model 3 0R (95% CI)	*P*-value
DII							
T1	63/8,773	Ref.	-	Ref.	-	Ref.	-
T2	78/8,773	1.24 (0.89–1.74)	0.206	1.26 (0.90–1.77)	0.180	1.22 (0.87–1.72)	0.244
T3	106/8.774	1.69 (1.24–2.32)	0.001	1.68 (1.22–2.33)	0.001	1.58 (1.13–2.21)	0.007
Continuous	247/170,334	1.31 (1.12–1.53)	< 0.001	1.30 (1.11–1.53)	0.001	1.26 (1.07–1.49)	0.007
DOBS							
T1	63/8,773	Ref.	-	Ref.	-	Ref.	-
T2	78/8,773	0.64 (0.47–0.86)	0.003	0.67 (0.49–0.90)	0.010	0.69 (0.50–0.94)	0.019
T3	106/8.774	0.66 (0.49–0.89)	0.007	0.78 (0.56–1.07)	0.122	0.83 (0.60–1.15)	0.257
Continuous	247/170,334	0.80 (0.68–0.93)	0.004	0.87 (0.73–1.02)	0.088	0.90 (0.76–0.99)	0.007

*Notes* DII, Dietary Inflammatory Index; DOBS, Dietary Oxidative Balance Score; OR, Odds ratio; CI, confidence interval; Model 1: No covariates were adjusted; Model 2: Adjusted for age, sex and race/ethnicity; Model 3: Adjusted for age, sex, race/ethnicity, education level, drinking status, smoking status, PIR, Hypertension and Diabetes

In Model 2, compared to the lowest tertile of DOBS, the second tertile showed a 33% reduced risk of GI cancers (OR 0.67, 95% CI 0.49 to 0.90, *P* = 0.010). In Model 3, a continuous DOBS was significantly negatively associated with the risk of GI cancers (OR 0.90, 95% CI 0.76 to 0.99, *P* = 0.007). Compared to the lowest tertile of DOBS, the second tertile showed a 31% reduced risk of GI cancers (OR 0.69, 95% CI 0.50 to 0.94, *P* = 0.019). For more details, see Table 2.

### Nonlinearity analysis using RCS

**Fig. 2:**
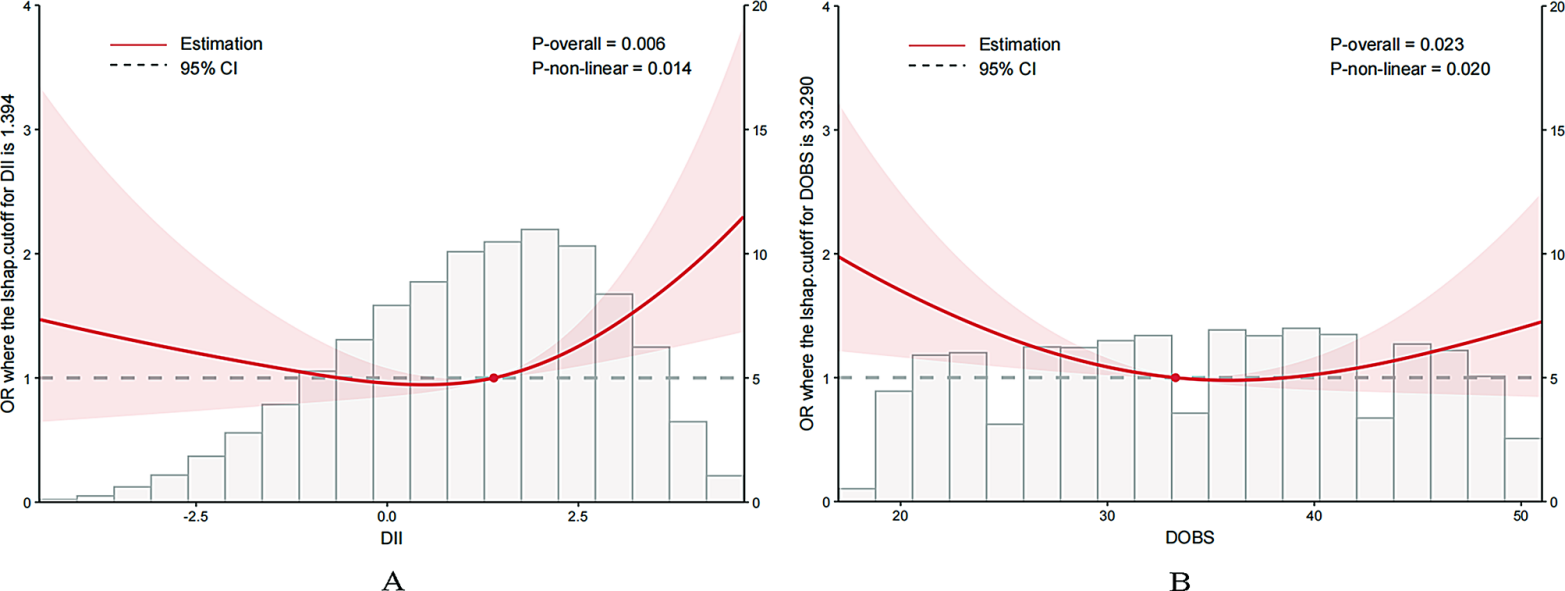
Association Between DII, DOBS and Gastrointestinal Cancers Using a Restricted Cubic Spline Regression Model. Legends: **A**), DII, Dietary Inflammatory Index; **B**), DOBS, Dietary Oxidative Balance Score. Graphs show ORs for end according to DII, DOBS adjusted for sex, age, race/ethnicity, education level, drinking status, smoking status, PIR, Hypertension, Diabetes. Data were fitted by a logistic regression model, and the model was conducted with 4 knots at the 5th, 35th, 65th, 95th percentiles of DII, DOBS (reference is the 5th percentile). Solid lines indicate ORs, and shadow shape indicate 95% CIs. OR, odds ratio; CI, confidence interval

A RCS model was used to evaluate the dose-response relationship between DII, DOBS, and GI cancers. The model included knots at the 5th, 35th, 65th, and 95th percentiles of continuous DII and DOBS values, with the 5th percentile as the reference. The model was adjusted for age, sex, and ethnicity. As shown in Fig. 2A, there was a significant nonlinear association between DII and GI cancers, with the curve exhibiting a U-shape. The lowest point was reached between 0 and 2 in continuous DII, then it gradually increased approaching 1 (p for overall association = 0.006, p for nonlinear association = 0.014). As shown in Fig. 2B, there was a significant nonlinear association between DOBS and GI cancers (p for overall association = 0.023, p for nonlinear association = 0.020). Figure 2 also illustrates the distributions of DII and DOBS in the study population. DII is right-skewed, ranging primarily from − 3 to 4, while DOBS also shows a right-skewed distribution, concentrated between 15 and 50. These patterns align with the results of the Shapiro-Wilk test (DII: *p* < 0.001; DOBS: *p* < 0.001), indicating deviations from normality.

### Mediation analysis

**Fig. 3:**
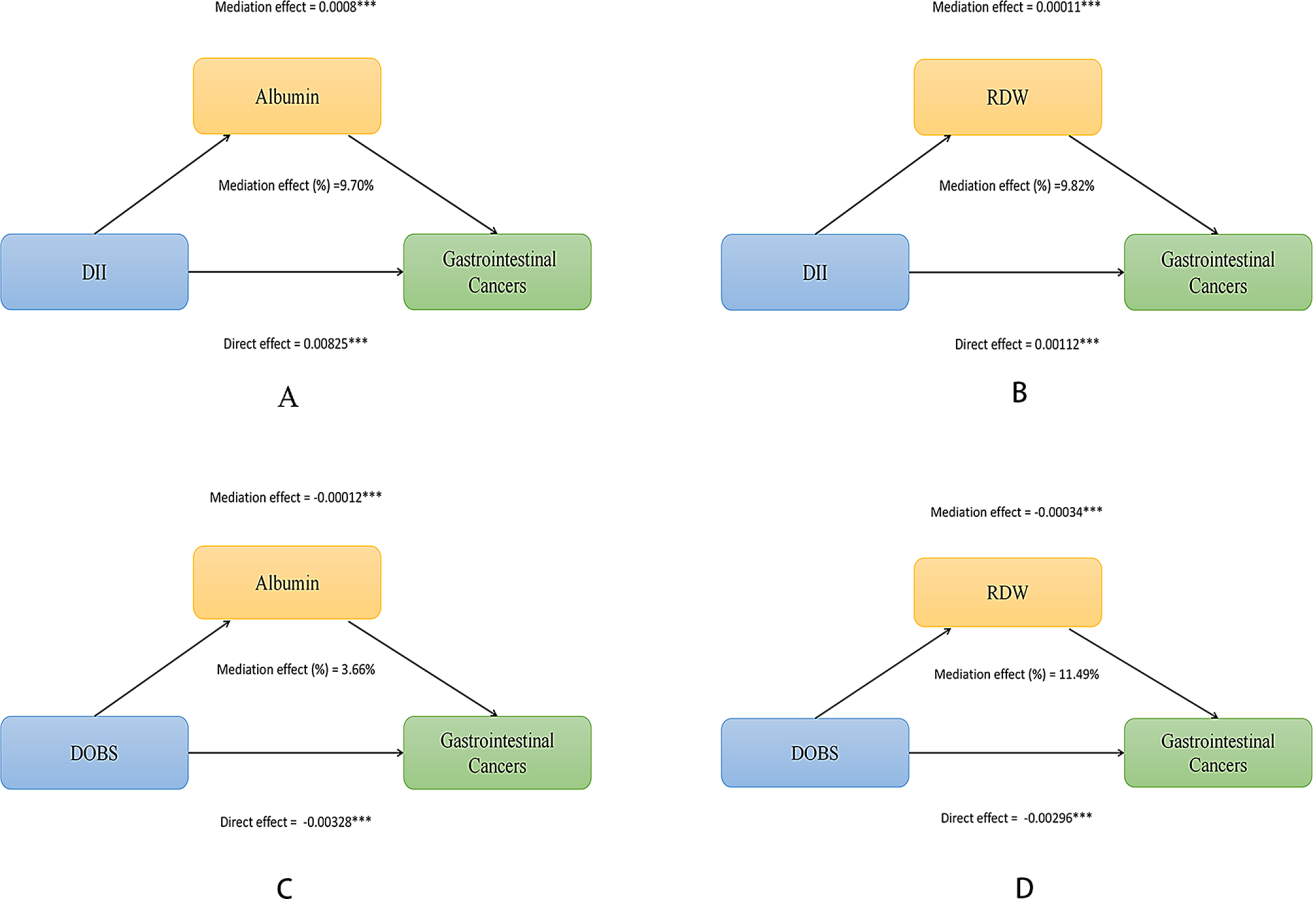
Mediation Analyses of the Association between DII, DOBS and Gastrointestinal Cancers. Legends: **A**), serum albumin, **B**), RDW partially mediates the relationship between DII and Gastrointestinal Cancers; **C**), serum albumin, **D**), RDW partially mediates the relationship between DOBS and Gastrointestinal Cancers

We constructed mediation analysis models to evaluate whether serum albumin and RDW mediate the relationship between DII, DOBS, and GI cancers. In these models, DII and DOBS were the independent variables, GI cancers the dependent variable, and serum albumin and RDW as mediators. As shown in Fig. 3A, serum albumin levels significantly mediated the association between DII and GI cancers, accounting for 9.70% of the association, with an IE of 0.0008 (95% CI: 0.0005 to 0.0012). As shown in Fig. 3B, RDW levels significantly mediated the association between DII and GI cancers, accounting for 9.82% of the association, with an IE of 0.00011 (95% CI: 0.00004 to 0.00017). As shown in Fig. 3C, serum albumin levels significantly mediated the association between DOBS and GI cancers, accounting for 3.66% of the association, with an IE of -0.00012 (95% CI: -0.00020 to -0.00005). As shown in Fig. 3D, RDW levels significantly mediated the association between DOBS and GI cancers, accounting for 11.49% of the association, with an IE of -0.00034 (95% CI: -0.00047 to -0.00021). More details can be seen in Fig. 3.

### Subgroup analysis and interaction

**Fig. 4:**
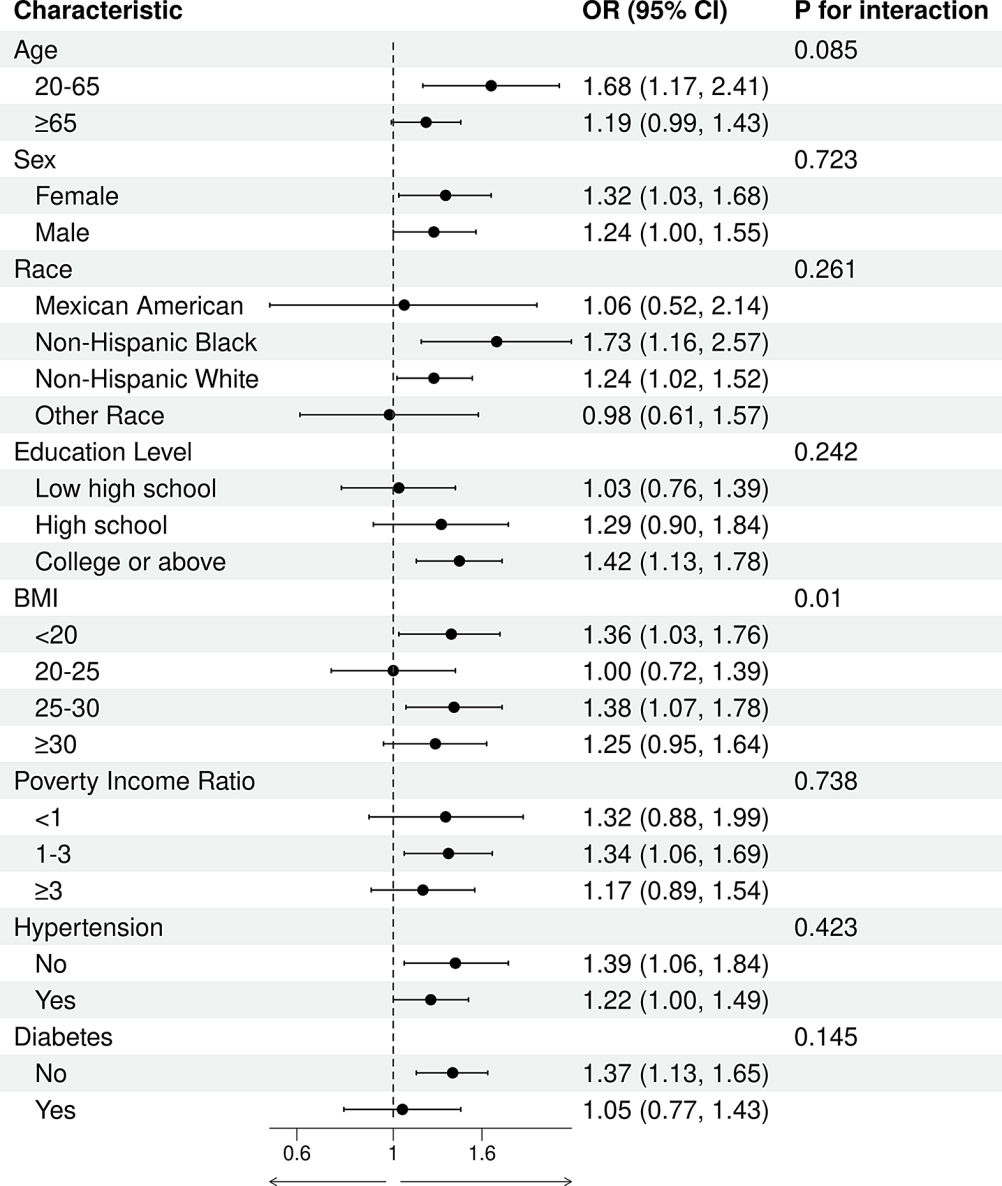
Subgroup analyses of the association between DII and gastrointestinal cancers

**Fig. 5:**
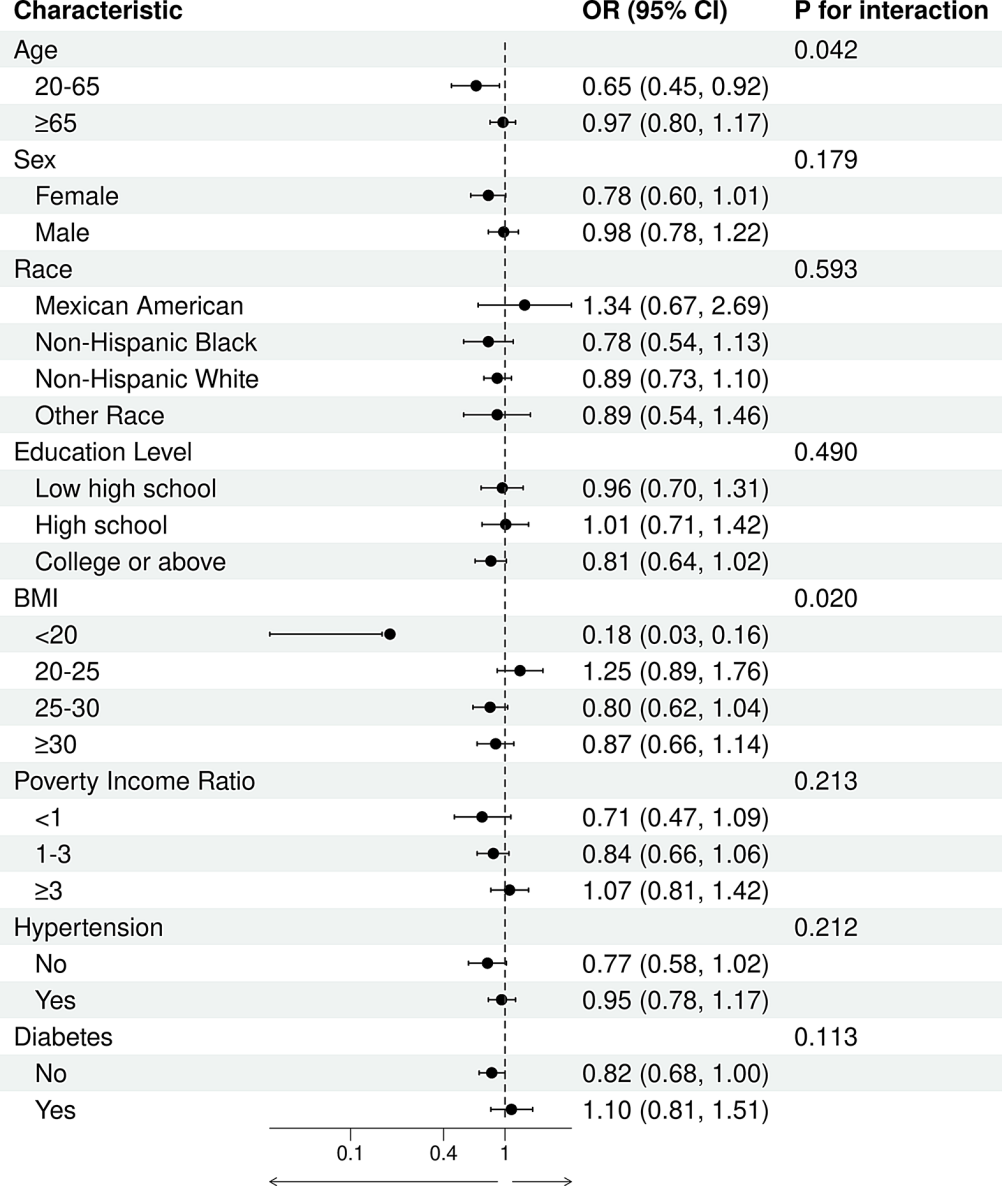
Subgroup analyses of the association between DOBS and gastrointestinal cancers

This study conducted subgroup analyses based on age, sex, ethnicity, educational level, BMI, PIR, hypertension, and diabetes. As shown in Fig. 4, DII was positively associated with the risk of GI cancers. This positive association was more pronounced among individuals aged 20–60 years(OR 1.68, 95%CI (1.17 to 2.41) ,females (OR 1.32, 95%CI (1.03 to 1.68), non-Hispanic Blacks (OR 1.73, 95%CI (1.16 to 2.57), those with a college education or higher (OR 1.42, 95%CI (1.13 to 1.78), individuals with a BMI between 25 and 30 (OR 1.38, 95%CI (1.07 to 1.78), those with a PIR between 1 and 3 (OR 1.34, 95%CI (1.06 to 1.69), individuals without hypertension (OR 1.39, 95%CI (1.06 to 1.84), and those without diabetes (OR 1.37, 95%CI (1.13 to 1.65). Interaction analyses showed significant interactions between DII and BMI. As shown in Fig. 5, DOBS was negatively associated with the risk of GI cancers. This negative correlation was more evident among individuals aged 20–60 years (OR 0.65, 95%CI (0.45 to 0.92) and those without diabetes. Interaction results indicated significant interactions between DOBS and both age and BMI.

### Spearman correlation analysis

As shown in Supplementary Fig. 3, the Spearman correlation analysis demonstrated a strong negative correlation between DII and DBOS (Spearman correlation coefficient = -0.8505, *p* < 0.001), indicating that as DII decreases (suggesting an anti-inflammatory diet), DBOS increases (suggesting an antioxidant diet). The scatter plot shows this relationship clearly, with the regression line highlighting the strong inverse association between these two scores. This analysis confirms that there are virtually no participants with both low DII and low DBOS, underscoring the consistent alignment of anti-inflammatory and antioxidant dietary characteristics.

## Discussion

This study utilized data from the NHANES database for the years 2005–2018, employing 28 dietary components associated with inflammatory potential to construct the DII, and used 17 nutrients related to oxidative balance, based on prior experience, to build the DOBS. These indexes represented participants’ pro-inflammatory and antioxidative dietary levels, respectively. Additionally, the relationship between these composite scores and GI cancers (including esophageal, gastric, liver, pancreatic, colon, and rectal cancers) was evaluated. Our findings indicated that in both univariate and multivariate logistic regression models, a higher DII was associated with an increased risk of GI cancers, while a higher DBOS was consistently associated with a reduced risk of these cancers. This suggests that both dietary factors, when aligned towards anti-inflammatory and antioxidant properties, are protective against GI cancer risk. RCS results revealed a significant nonlinear dose-response relationship between DII, DOBS and GI cancers. Mediation analysis demonstrated that serum albumin and RDW partially mediated the association between DII, DOBS, and GI cancers. The effects of DII and DOBS on the risk of GI cancers were consistent across subgroups by sex, ethnicity, age, educational level, BMI, hypertension, and diabetes, adding stability and reliability to the conclusions.

This study is among the first to specifically combine the analysis of anti-inflammatory and antioxidant capacities in dietary components with the risk of GI cancers, examining their joint mediating roles in this relationship. Our findings are largely consistent with previous research and extend earlier discoveries regarding the relationship between pro-inflammatory diets, antioxidant diets, and GI cancers. Some cross-sectional and prospective studies indicate that pro-inflammatory diets (high in fats and sugars) induce chronic low-grade inflammation, thereby promoting the development of GI cancers [18]. A recent systematic review by Chen et al. suggests that individuals with higher DII scores have a 2.16-fold increased relative risk of liver cancer [19]. Western dietary patterns, including high intake of red meat, high-fat dairy products, refined grains, and simple carbohydrates, are closely linked to a high incidence of colorectal cancer [20]. Intake of vitamin E, unsaturated fatty acids, and high-fiber diets can reduce the incidence of tumors, including GI cancers [21–23]. Our study not only confirms these associations but also demonstrates the link between DII, DOBS, and the risk of GI cancers in a nationally representative cohort. These studies support our findings and highlight the importance of anti-inflammatory and antioxidant diets in the prevention and management of GI cancers.

The following mechanisms may further explain these findings: A high DII score reflects a diet with higher pro-inflammatory levels, and chronic inflammation is a well-established cause of cancer due to inflammatory cytokines like TNF-α, IL-6, and IL-1β causing DNA damage, abnormal DNA methylation, and activating NF-κB, leading to oncogene activation and tumor suppressor gene inactivation [24–26]. The definition of OBS varies, with different scoring schemes including various pro-oxidants and antioxidants [27]. Generally, a lower OBS, indicating higher oxidative stress, negatively impacts health. This study included 14 antioxidants and 3 pro-oxidants. Oxidative stress, involving reactive oxygen species (ROS) and reactive nitrogen species (RNS), leads to cellular damage through lipid peroxidation, protein modification, and DNA damage [28], causing genetic and epigenetic alterations linked to cancer progression. Key lipid peroxidation by-products, malondialdehyde (MDA) and 4-hydroxynonenal (4-HNE), are toxic, mutagenic, and can cause extensive cellular damage [29], and have been linked to aging, neurodegenerative diseases, and cancer, with elevated levels found in primary colorectal cancer [30]. ROS also oxidize protein residues, altering structure and function. Helicobacter pylori infection, a critical factor in gastritis, induces ROS release from neutrophils, which, processed by myeloperoxidase, forms the oxidant hypochlorite anion. This reacts with ammonia produced by Helicobacter pylori urease to generate monochloramine (NH2Cl), which oxidizes intracellular components, causing damage [26]. Persistent DNA damage and repair failures contribute to genomic instability, promoting cancer development and progression [31, 32].

Our mediation analysis suggests that serum albumin and RDW partially mediate the relationship between DII, DOBS, and GI cancers. Serum albumin reflects nutritional status and is associated with systemic inflammation in tumors. Malnutrition and inflammation can inhibit albumin synthesis, correlating with cancer prognosis [33, 34]. Albumin has anticancer functions, such as scavenging free radicals and stabilizing DNA replication [35], and higher oxidative stress accelerates albumin consumption due to increased free radical production [36]. Consequently, higher oxidative stress is linked to lower serum albumin levels, which is a prognostic factor for survival in various cancers, including colorectal [37], pancreatic [38], and gastric cancers [39]. Albumin’s physiological anticancer effects include its antioxidant properties and DNA replication stabilization [40]. Proposed mechanisms for low albumin in cancer patients include the production of inflammatory cytokines like IL-6 and increased vascular permeability, which raises the transcapillary escape rate of albumin, reducing serum levels [41, 42].

RDW reflects red blood cell size variability, or anisocytosis, and has been linked to inflammation and oxidative stress in various conditions, including cardiovascular diseases, venous thromboembolism, and cancer [43]. Research has demonstrated that RDW can reflect systemic anti-inflammatory responses, and the presence, invasion, metastasis, and recurrence of tumors [12]. Besides, elevated RDW is closely linked to oxidative stress, which increases red cell membrane fragility, shortens lifespan, and enhances red cell heterogeneity. Thus, RDW reflects both systemic inflammation and increased oxidative stress levels [44, 45]. In addition, RDW has been studied as a prognostic factor in cancers, such as colorectal cancer, where elevated preoperative RDW predicts poor outcomes [46, 47]. Mechanisms linking elevated RDW to poor prognosis in cancer include inflammation suppressing erythropoietin activity and increased oxidative stress reducing red cell lifespan and stability [48, 49]. Insufficient intake of nutrients and micronutrients, particularly hematopoietic raw materials like vitamin B12, folate, and iron, suppresses red blood cell production and alters the deformability of red cell membranes, leading to increased RDW levels [50]. Increasing evidence suggests that RDW may serve as a diagnostic or prognostic biomarker in various solid cancers [44]. Further prospective and larger-scale studies are needed to determine the role of RDW as an early biological marker in the diagnosis or activity of cancer.

Although the mediation effects of 4% through serum albumin and around 10% through RDW may appear modest, they could still hold clinical significance, particularly given the complex interplay of dietary factors in disease pathogenesis. Even small mediation effects can meaningfully influence GI cancer risk, especially in high-incidence populations, potentially informing targeted prevention and intervention strategies [51]. The 10% effect via RDW, in particular, may reflect a clinically relevant pathway, suggesting that dietary factors influence GI cancer development through mechanisms like inflammation and oxidative stress [52]. Future research should investigate these pathways further to clarify their role across diverse populations.

While previous studies have demonstrated that diet influences the incidence of GI cancers, our research adds several new strengths to the existing literature: Firstly, based on prior experience, we constructed two comprehensive dietary indices, DII and DOBS, to assess levels of dietary inflammation and oxidative stress, focusing on the combined effects of multiple dietary components compared to the impact of single dietary factors on GI cancers. Secondly, we conducted subgroup analyses, which indicated significant statistical differences in the impact of DOBS on GI tumor risk across age and BMI subgroups, highlighting the importance of considering age and BMI-specific factors in the research and management of GI cancers.

Additionally, we explored the potential mediating roles of serum albumin and RDW in the relationship between DII, DOBS, and GI cancers. To our knowledge, this is the first study to explore these factors as potential mediators in this context. Our results from the RCS model suggest a significant nonlinear dose-response relationship between DII, DOBS and GI cancers, underscoring the complexity of dietary inflammation and oxidative stress in the pathophysiology of GI cancers and highlighting the importance of maintaining optimal dietary inflammation balance [20]. This finding has significant implications for personalized dietary interventions in the prevention and management of GI cancers. Finally, we utilized a large, nationally representative sample from the NHANES database, which strengthens the generalizability of our findings to the U.S. population.

This study has certain limitations; first, it is a cross-sectional study, which constrains the exploration of the causal relationships between DII, DOBS, and GI cancers. Additionally, the dietary quality assessment data in this study were derived from participants’ self-reported 24-hour recall questionnaires, which may lead to inaccuracies in conclusions due to recall bias among participants. Furthermore, although we adjusted for age, gender, race, education level, alcohol consumption, smoking status, PIR, physical activity, hypertension, diabetes, and other factors in the multivariable logistic regression models, there remain unmeasured or unknown confounding factors that may affect the relationship between dietary quality and gastrointestinal tumors.

For future researches, we recommend conducting large-scale, longitudinal cohort studies to establish temporal relationships and better understand the causality between dietary inflammation, oxidative stress, and GI cancers. Specifically, future studies should aim to identify the critical time windows during which dietary inflammation and oxidative stress most significantly impact cancer risk. Furthermore, it is important to investigate dose-response relationships between DII, DOBS, and GI cancer risk across diverse populations, including those with varying genetic backgrounds and lifestyle factors. To delve deeper into the biological mechanisms, integrating dietary data with multi-omics approaches—such as genomics, metabolomics, and transcriptomics—can help uncover specific pathways linking diet to cancer pathogenesis.

In addition, developing and testing specific dietary interventions that target the reduction of dietary inflammation and oxidative stress should be prioritized, especially in clinical trials to assess their efficacy in reducing GI cancer risk. Exploring the interactions between dietary factors and other modifiable risk factors, such as physical activity, will also provide a more comprehensive understanding of how lifestyle modifications can synergistically reduce GI cancer risk. By addressing these specific areas, future research can effectively elucidate the underlying mechanisms and inform personalized dietary strategies for the prevention and management of GI cancers.

## Conclusions

In summary, this study utilized a large, nationally representative sample from NHANES to investigate the relationship between dietary inflammation and oxidative stress, as assessed by DII and DOBS, and the risk of GI cancers. The study revealed a significant positive correlation between higher DII and increased incidence of GI cancers, and a significant negative correlation between higher DOBS and increased incidence of GI cancers. Additionally, the study identified serum albumin and RDW as important mediators in the relationship between DII, DOBS, and GI cancers. Therefore, advocating for a healthy diet is crucial for maintaining homeostasis and improving human quality of life. Changes in diet and lifestyle can alter the risk of gastrointestinal cancers; however, since specific nutrients are not consumed in isolation but as part of dietary patterns with interactive components, future research should increasingly consider multiple dietary components as a whole to assess their impact on cancer progression and thereby develop individualized and rational dietary strategies for the dietary management and prevention of GI cancers.

## Electronic supplementary material

Below is the link to the electronic supplementary material.

Supplementary Material 1

## Data Availability

This study used data from the National Health and Nutrition Examination Survey (NHANES) (https://www.cdc.gov/nchs/nhanes/index.htm).
