# Supplementary material for: Association of dietary inflammatory index and dietary oxidative balance score with gastrointestinal cancers in NHANES 2005–2018
Source: BMC Public Health. 2024 Oct 9;24:2760. doi: 10.1186/s12889-024-20268-4 (PMC11465896; doi:10.1186/s12889-024-20268-4)

**Title: Association of Dietary Inflammatory Index and Dietary Oxidative Balance Score with Gastrointestinal Cancers in NHANES 2005-2018**

**Supplementary Tables 1-2 (included in this file)**

**Supplementary Table 1.** Dietary composition parameters involved in DII, inflammatory effect scores, and intake values from the global composite data setd

| Dietary | Raw | Overall | Global daily | Standard |
| --- | --- | --- | --- | --- |
| composition | inflammatory | inflammatory | mean intake | deviation of |
| parameter | effect scoreb | effect scorec | (units/d) | the global daily intake |
| Alcohol (g) | -0.277 | -0.277 | 13.98 | 3.72 |
| Vitamin B12 (μg) | 0.205 | 0.106 | 5.15 | 2.70 |
| Vitamin B6 (mg) | -0.379 | -0.365 | 1.47 | 0.74 |
| β-Carotene (μg) | -0.584 | -0.584 | 3718 | 1720 |
| Caffeine (g) | -0.124 | -0.110 | 8.05 | 6.67 |
| Carbohydrate (g) | 0.109 | 0.097 | 272.2 | 40 |
| Cholesterol (mg) | 0.347 | 0.110 | 279.4 | 51.2 |
| Energy (kcal) | 0.180 | 0.180 | 2056 | 338 |
| Total fat (g) | 0.298 | 0.298 | 71.4 | 19.4 |
| Fiber (g) | -0.663 | -0.663 | 18.8 | 4.9 |
| Folic acid (μg) | -0.207 | -0.190 | 273 | 70.7 |
| Iron (mg) | 0.032 | 0.032 | 13.35 | 3.71 |
| Magnesium (mg) | -0.484 | -0.484 | 310.1 | 139.4 |
| MUFAe (g) | -0.019 | -0.009 | 27 | 6.1 |
| Niacin (mg) | -1.000 | -0.246 | 25.9 | 11.77 |
| Protein (g) | 0.049 | 0.021 | 79.4 | 13.9 |
| PUFAf (g) | -0.337 | -0.337 | 13.88 | 3.76 |
| Vitamin B2 (mg) | -0.727 | -0.068 | 1.7 | 0.79 |
| Saturated fat (g) | 0.429 | 0.373 | 28.6 | 8 |
| Selenium (μg) | -0.191 | -0.191 | 67 | 25.1 |
| Vitamin B1 (mg) | -0.354 | -0.098 | 1.7 | 0.66 |
| Vitamin A (REa) | -0.401 | -0.401 | 983.9 | 518.6 |
| Vitamin C (mg) | -0.424 | -0.424 | 118.2 | 43.46 |
| Vitamin D (μg) | -0.446 | -0.446 | 6.26 | 2.21 |
| Vitamin E (mg) | -0.419 | -0.419 | 8.73 | 1.49 |
| Zinc (mg) | -0.313 | -0.313 | 9.84 | 2.19 |

aRetinol equivalents.

bDietary composition parameter-specific raw inflammatory effect score, which is per unit amount noted for each food parameter.

cDietary composition parameter-specific overall inflammatory effect score.

dDII of a certain dietary component = (Daily intake of the dietary component - Global daily mean intake of the dietary component) / Standard deviation of the global daily intake for the dietary component * Overall inflammatory effect score of the dietary component. The DII for each participant was obtained by summing the DII of the 26 dietary components selected in this study.

eMonounsaturated fatty acids. fPolyunsaturated fatty acids.

**Supplementary Table 2.** DOBS components and score

| DOBS | Property | DOBS Score | | |
| --- | --- | --- | --- | --- |
|  |  | 1 | 2 | 3 |
| Dietary fiber (g/d) | Aa | ≤11.95 | 11.96-18.60 | >18.60 |
| Ln-transformed carotene (μg/d) | A | ≤8.31 | 8.32-9.20 | >9.21 |
| Vitamin B2 (mg/d) | A | ≤1.53 | 1.54-2.25 | >2.25 |
| Niacin (mg/d) | A | ≤18.50 | 18.51-27.24 | >27.24 |
| Vitamin B6 (mg/d) | A | ≤1.46 | 1.47-2.38 | >2.39 |
| Folic acid (μg/d) | A | ≤100.50 | 100.51-192.00 | >192.00 |
| Ln-transformed vitamin B12 (μg/d) | A | ≤0.94 | 0.95-1.21 | >1.22 |
| Ln-transformed vitamin C (mg/d) | A | ≤3.87 | 3.88-4.67 | >4.68 |
| Vitamin E (mg/d) | A | ≤5.40 | 5.41-8.57 | >8.57 |
| Calcium (mg/d) | A | ≤662.50 | 662.51-1016.50 | >1016.51 |
| Magnesium (mg/d) | A | ≤224.00 | 224.01-314.00 | >314.01 |
| Zinc (mg/d) | A | ≤9.06 | 9.07-12.86 | >12.87 |
| Copper (mg/d) | A | ≤0.99 | 1.00-1.47 | >1.48 |
| Selenium (μg/d) | A | ≤83.60 | 83.61-120.90 | >120.91 |
| Total fat (g/d) | Pb | >84.94 | 56.55-84.93 | ≤56.54 |
| Iron (mg/d) | P | >16.53 | 11.44-16.52 | ≤11.43 |
| Alcohol (male) (g/d) | P | >30.01 | 0.01-30.00 | 0.00 |
| Alcohol (female) (g/d) | P | >15.01 | 0.01-15.00 | 0.00 |

^a^Antioxidant.

^b^Prooxidant.

**Supplementary Table 3.** Distribution of Gastrointestinal Cancer Types in the Study Population

| GI Cancer Type | Number of Cases (n) |
| --- | --- |
| Total GI Cancers | 247 |
| Colon cancer | 184 |
| Esophageal cancer | 20 |
| Rectal cancer | 8 |
| Gastric cancer | 14 |
| Liver cancer | 17 |
| Pancreatic cancer | 4 |

**Supplementary Table 4.** Characteristics of the Study Population by DII Level

| Characteristic |  | DII | | | |
| --- | --- | --- | --- | --- | --- |
|  | Overall, N = 26,3201 | T1, N = 8,773 | T2, N = 8,773 | T3, N = 8,774 | p-value |
| Sex |  |  |  |  | <0.001 |
| Male | 12,722 (48%) | 5,282 (60%) | 4,247 (48%) | 3,193 (36%) |  |
| Female | 13,598 (52%) | 3,491 (40%) | 4,526 (52%) | 5,581 (64%) |  |
| Age |  |  |  |  | 0.034 |
| Mean (SD) | 50 (18) | 50 (17) | 49 (18) | 50 (18) |  |
| BMI (kg/m2) |  |  |  |  | <0.001 |
| <20 | 1,142 (4.3%) | 388 (4.4%) | 346 (3.9%) | 408 (4.7%) |  |
| 20-25 | 6,247 (24%) | 2,396 (27%) | 1,993 (23%) | 1,858 (21%) |  |
| 25-30 | 8,654 (33%) | 3,075 (35%) | 2,927 (33%) | 2,652 (30%) |  |
| ≥30 | 10,277 (39%) | 2,914 (33%) | 3,507 (40%) | 3,856 (44%) |  |
| Education Level (%) |  |  |  |  | <0.001 |
| Low high school | 5,807 (22%) | 1,403 (16%) | 1,917 (22%) | 2,487 (28%) |  |
| High school | 6,083 (23%) | 1,649 (19%) | 2,080 (24%) | 2,354 (27%) |  |
| College or above | 14,430 (55%) | 5,721 (65%) | 4,776 (54%) | 3,933 (45%) |  |
| Race/ethnicity (%) |  |  |  |  | <0.001 |
| Mexican American | 3,847 (15%) | 1,334 (15%) | 1,364 (16%) | 1,149 (13%) |  |
| Non-Hispanic Black | 5,566 (21%) | 1,366 (16%) | 1,884 (21%) | 2,316 (26%) |  |
| Non-Hispanic White | 12,145 (46%) | 4,311 (49%) | 3,988 (45%) | 3,846 (44%) |  |
| Other Race | 4,762 (18%) | 1,762 (20%) | 1,537 (18%) | 1,463 (17%) |  |
| Drinking status (%) |  |  |  |  | <0.001 |
| Yes | 7,248 (28%) | 1,948 (22%) | 2,374 (27%) | 2,926 (33%) |  |
| No | 19,072 (72%) | 6,825 (78%) | 6,399 (73%) | 5,848 (67%) |  |
| Smokers |  |  |  |  | <0.001 |
| Yes | 14,335 (54%) | 4,979 (57%) | 4,882 (56%) | 4,474 (51%) |  |
| No | 11,985 (46%) | 3,794 (43%) | 3,891 (44%) | 4,300 (49%) |  |
| Poverty Income Ratio (%) |  |  |  |  | <0.001 |
| <1 | 5,245 (20%) | 1,271 (14%) | 1,690 (19%) | 2,284 (26%) |  |
| 1-3 | 11,008 (42%) | 3,302 (38%) | 3,700 (42%) | 4,006 (46%) |  |
| ≥3 | 10,067 (38%) | 4,200 (48%) | 3,383 (39%) | 2,484 (28%) |  |
| Physical activity (%) |  |  |  |  | <0.001 |
| Moderate | 14,460 (55%) | 4,521 (52%) | 4,796 (55%) | 5,143 (59%) |  |
| Vigorous | 6,179 (23%) | 2,154 (25%) | 2,048 (23%) | 1,977 (23%) |  |
| Inactive | 5,681 (22%) | 2,098 (24%) | 1,929 (22%) | 1,654 (19%) |  |
| Hypertension (%) |  |  |  |  | <0.001 |
| No | 16,700 (63%) | 5,853 (67%) | 5,604 (64%) | 5,243 (60%) |  |
| Yes | 9,620 (37%) | 2,920 (33%) | 3,169 (36%) | 3,531 (40%) |  |
| Diabetes (%) |  |  |  |  | <0.001 |
| No | 22,921 (87%) | 7,832 (89%) | 7,614 (87%) | 7,475 (85%) |  |
| Yes | 3,399 (13%) | 941 (11%) | 1,159 (13%) | 1,299 (15%) |  |
| Albumin (g/dl) |  |  |  |  | <0.001 |
| Mean (SD) | 4.21 (0.36) | 4.27 (0.35) | 4.21 (0.36) | 4.16 (0.35) |  |
| Missing | 1,129 | 299 | 393 | 437 |  |
| RDW |  |  |  |  | <0.001 |
| Mean (SD) | 13.30 (1.36) | 13.13 (1.20) | 13.29 (1.35) | 13.47 (1.49) |  |
| Missing | 814 | 223 | 290 | 301 |  |

Notes: DII, Dietary Inflammatory Index; SD, Standard Deviation; BMI, Body Mass Index; RDW, Red Cell Distribution Width;

**Supplementary Table 5.** Characteristics of the Study Population by DOBS Level

| Characteristic |  | DOBS | | | |
| --- | --- | --- | --- | --- | --- |
|  | Overall, N = 26,3201 | T1, N = 8,543 | T2, N = 8,931 | T3, N = 8,846 | p-value |
| Sex |  |  |  |  | <0.001 |
| Male | 12,722 (48%) | 2,758 (32%) | 4,084 (46%) | 5,880 (66%) |  |
| Female | 13,598 (52%) | 5,785 (68%) | 4,847 (54%) | 2,966 (34%) |  |
| Age |  |  |  |  | <0.001 |
| Mean (SD) | 50 (18) | 52 (18) | 50 (18) | 48 (17) |  |
| BMI (kg/m2) |  |  |  |  | <0.001 |
| <20 | 1,142 (4.3%) | 393 (4.6%) | 370 (4.1%) | 379 (4.3%) |  |
| 20-25 | 6,247 (24%) | 1,876 (22%) | 2,109 (24%) | 2,262 (26%) |  |
| 25-30 | 8,654 (33%) | 2,665 (31%) | 2,949 (33%) | 3,040 (34%) |  |
| ≥30 | 10,277 (39%) | 3,609 (42%) | 3,503 (39%) | 3,165 (36%) |  |
| Education Level (%) |  |  |  |  | <0.001 |
| Low high school | 5,807 (22%) | 2,428 (28%) | 1,889 (21%) | 1,490 (17%) |  |
| High school | 6,083 (23%) | 2,203 (26%) | 2,013 (23%) | 1,867 (21%) |  |
| College or above | 14,430 (55%) | 3,912 (46%) | 5,029 (56%) | 5,489 (62%) |  |
| Race/ethnicity (%) |  |  |  |  | <0.001 |
| Mexican American | 3,847 (15%) | 1,204 (14%) | 1,268 (14%) | 1,375 (16%) |  |
| Non-Hispanic Black | 5,566 (21%) | 2,328 (27%) | 1,813 (20%) | 1,425 (16%) |  |
| Non-Hispanic White | 12,145 (46%) | 3,493 (41%) | 4,197 (47%) | 4,455 (50%) |  |
| Other Race | 4,762 (18%) | 1,518 (18%) | 1,653 (19%) | 1,591 (18%) |  |
| Drinking status (%) |  |  |  |  | <0.001 |
| Yes | 7,248 (28%) | 2,864 (34%) | 2,461 (28%) | 1,923 (22%) |  |
| No | 19,072 (72%) | 5,679 (66%) | 6,470 (72%) | 6,923 (78%) |  |
| Smokers |  |  |  |  | 0.008 |
| Yes | 14,335 (54%) | 4,539 (53%) | 4,947 (55%) | 4,849 (55%) |  |
| No | 11,985 (46%) | 4,004 (47%) | 3,984 (45%) | 3,997 (45%) |  |
| Poverty Income Ratio (%) |  |  |  |  | <0.001 |
| <1 | 5,245 (20%) | 2,182 (26%) | 1,662 (19%) | 1,401 (16%) |  |
| 1-3 | 11,008 (42%) | 3,831 (45%) | 3,713 (42%) | 3,464 (39%) |  |
| ≥3 | 10,067 (38%) | 2,530 (30%) | 3,556 (40%) | 3,981 (45%) |  |
| Physical activity (%) |  |  |  |  | <0.001 |
| Moderate | 14,460 (55%) | 5,193 (61%) | 4,912 (55%) | 4,355 (49%) |  |
| Vigorous | 6,179 (23%) | 1,883 (22%) | 2,129 (24%) | 2,167 (24%) |  |
| Inactive | 5,681 (22%) | 1,467 (17%) | 1,890 (21%) | 2,324 (26%) |  |
| Hypertension (%) |  |  |  |  | <0.001 |
| No | 16,700 (63%) | 5,023 (59%) | 5,704 (64%) | 5,973 (68%) |  |
| Yes | 9,620 (37%) | 3,520 (41%) | 3,227 (36%) | 2,873 (32%) |  |
| Diabetes (%) |  |  |  |  | <0.001 |
| No | 22,921 (87%) | 7,232 (85%) | 7,788 (87%) | 7,901 (89%) |  |
| Yes | 3,399 (13%) | 1,311 (15%) | 1,143 (13%) | 945 (11%) |  |
| Albumin (g/dl) |  |  |  |  | <0.001 |
| Mean (SD) | 4.21 (0.36) | 4.16 (0.35) | 4.21 (0.35) | 4.26 (0.36) |  |
| Missing | 1,129 | 428 | 388 | 313 |  |
| RDW |  |  |  |  | <0.001 |
| Mean (SD) | 13.30 (1.36) | 13.50 (1.52) | 13.27 (1.33) | 13.13 (1.20) |  |
| Missing | 814 | 290 | 290 | 234 |  |

Notes: DOBS, Dietary Oxidative Balance Score; SD, Standard Deviation; BMI, Body Mass Index; RDW, Red Cell Distribution Width;

**Supplementary Figures 1-3**

**Supplementary Figure 1.** Directed Acyclic Graph (DAG) Illustrating the Relationship Between Dietary Inflammatory Index Exposure, Covariates, and Gastrointestinal Cancers


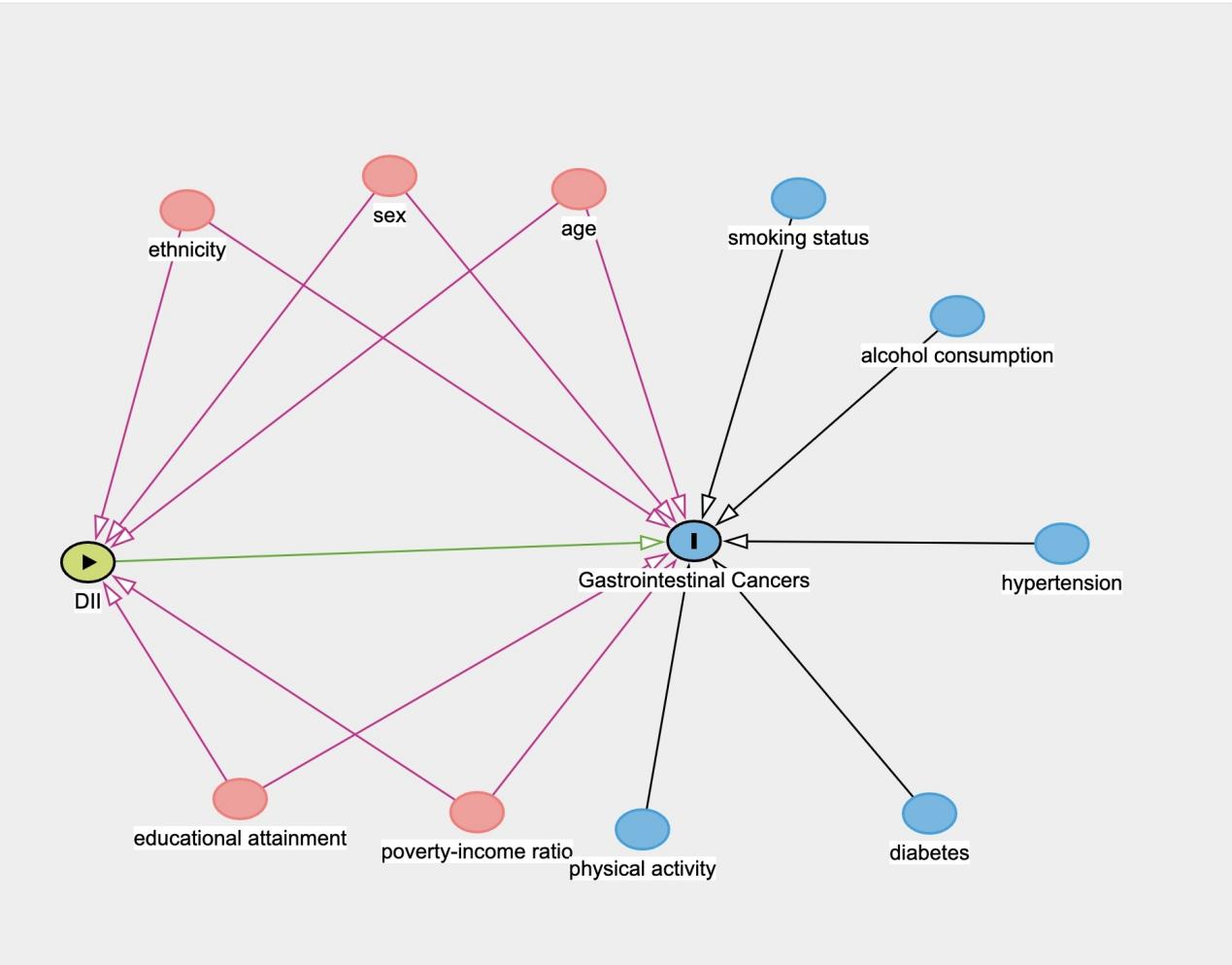


**Supplementary Figure 2**. Directed Acyclic Graph (DAG) Illustrating the Relationship Between Dietary Oxidative Balance Score Exposure, Covariates, and Gastrointestinal Cancers


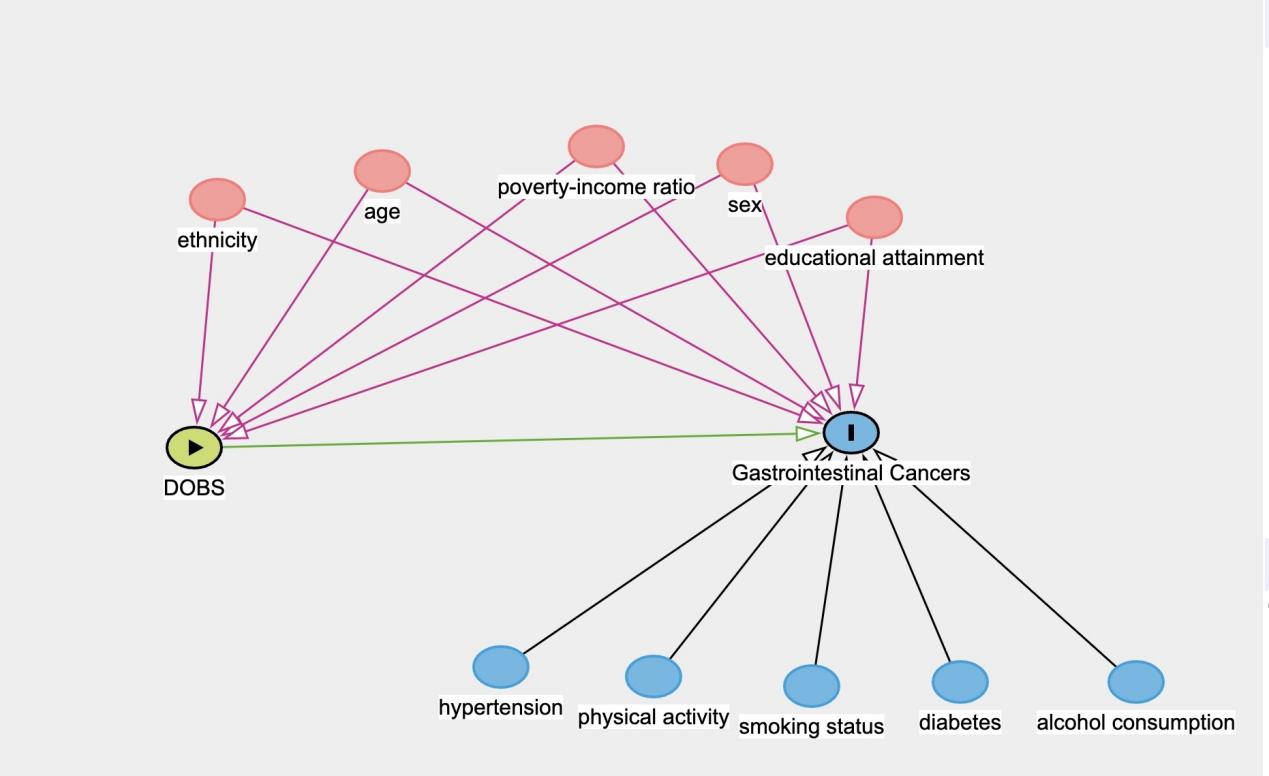


**Supplementary Figure 3.** Spearman correlation analysis demonstrated the correlation between Dietary Inflammatory Index (DII) and Dietary Oxidative Balance Score (DOBS)


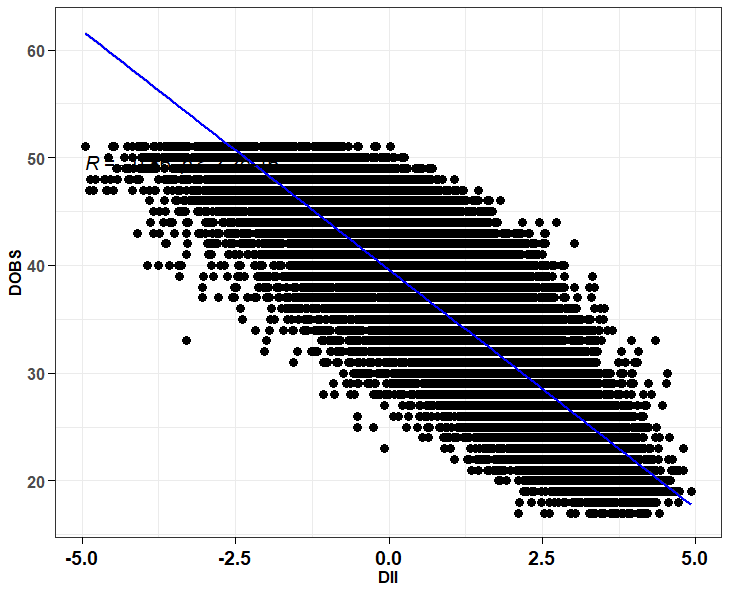

Supplement: Supplementary file 1 — Supplementary Material 1 [file 12889_2024_20268_MOESM1_ESM.docx]
